# Democracy-Independence Trade-Off in Oscillating Dendrites and Its Implications for Grid Cells

**DOI:** 10.1016/j.neuron.2010.04.027

**Published:** 2010-05-13

**Authors:** Michiel W.H. Remme, Máté Lengyel, Boris S. Gutkin

**Affiliations:** 1 Group for Neural Theory, Département d'Études Cognitives, École Normale Supérieure, 29 rue d'Ulm, 75005 Paris, France; 2 Computational and Biological Learning Lab, Department of Engineering, University of Cambridge, Trumpington Street, Cambridge CB2 1PZ, UK

**Keywords:** SYSNEURO

## Abstract

Dendritic democracy and independence have been characterized for near-instantaneous processing of synaptic inputs. However, a wide class of neuronal computations requires input integration on long timescales. As a paradigmatic example, entorhinal grid fields have been thought to be generated by the democratic summation of independent dendritic oscillations performing direction-selective path integration. We analyzed how multiple dendritic oscillators embedded in the same neuron integrate inputs separately and determine somatic membrane voltage jointly. We found that the interaction of dendritic oscillations leads to phase locking, which sets an upper limit on the timescale for independent input integration. Factors that increase this timescale also decrease the influence that the dendritic oscillations exert on somatic voltage. In entorhinal stellate cells, interdendritic coupling dominates and causes these cells to act as single oscillators. Our results suggest a fundamental trade-off between local and global processing in dendritic trees integrating ongoing signals.

## Highlights

► Biophysical properties predict phase locking of oscillating dendrites ► Trade-off between dendritic independence and democracy in oscillatory regime ► Integrating inputs on long timescales favors global dendritic processing ► Intradendritic oscillations are not sufficient to maintain grid fields

## Introduction

Dendritic trees possess a wide variety of voltage-dependent processes (Stuart et al., 2007) that render them into sophisticated computing devices (Poirazi et al., 2003). Previous studies characterized how synaptic inputs are mapped into dendritic membrane potentials, for example into dendritic spikes (Golding and Spruston, 1998), and how the local membrane potential signals from several such dendritic units then jointly determine the somatic membrane potential and ultimately the action potential output of cells (Rudolph and Destexhe, 2003). Two key features of neuronal computation emerged from these studies. First, the dendritic tree can consist of several functional compartments, each processing its inputs locally and largely independently from the other compartments (“dendritic independence,” Polsky et al., 2004). Second, the soma integrates the outputs of these compartments in a way such that even distant compartments exert an influence on it (“dendritic democracy,” Häusser, 2001). However, most previous studies considered only near-instantaneous processing of inputs in dendrites, whereby the membrane potential depends only on the recent 10–100 ms past of synaptic activity (Gasparini et al., 2004; Larkum et al., 2009; Losonczy and Magee, 2006; Nevian et al., 2007; Wei et al., 2001). Thus, it is still unclear how much dendritic independence and democracy can be maintained in the face of ongoing signals that require continuous integration on the timescale of seconds to minutes.

The issue of dendritic independence and democracy for ongoing processing lies at the heart of a prominent theory for the formation of grid fields in the entorhinal cortex. “Grid cells” of the rat medial entorhinal cortex respond by forming characteristic grid patterns of activation when the animal is navigating through its environment (Hafting et al., 2005; Sargolini et al., 2006). These hexagonal grid patterns remain stable over long periods of time and even persist in the dark for as long as 30 min (Hafting et al., 2005). This persistence is a signature of a path integration mechanism that computes the spatial position of the animal by the continuous integration of idiothetic cues (McNaughton et al., 2006). Intrinsic membrane potential oscillations have been proposed to be particularly well-suited for integrating synaptic inputs on long timescales (Huhn et al., 2005), and may thus play a key role in path integration (Lengyel et al., 2003). This is because the phase of an oscillator naturally integrates inputs modulating its frequency. Consequently, the single-cell theory that provides a mechanistic explanation for the firing pattern of grid cells posits the existence of several independent oscillatory units in the dendritic tree—each integrating the animal's velocity along a different direction—and a “democratic” summation of the signals contributed by these dendritic oscillations at the soma (Burgess et al., 2007; Hasselmo, 2007; O'Keefe and Burgess, 2005).

The “multiple oscillator” theory of grid cells is supported by several lines of evidence. First, entorhinal spiny stellate cells show subthreshold membrane potential oscillations (Alonso and Klink, 1993; Alonso and Llinás, 1989), which appear to result from the interaction between a persistent sodium current and the hyperpolarization-activated inward current (Alonso and Llinás, 1989; Dickson et al., 2000; Fransén et al., 2004; Rotstein et al., 2006). Second, the theory not only reproduces the hexagonally tessellated firing rate fields of grid cells, but it can also successfully account for the gradual precession of the timing of grid cell firings relative to the local field theta oscillation as the animal passes through each peak of the grid field (Hafting et al., 2008). Finally, the theory can also account for the correlation between the frequencies of intrinsic oscillations and the spacing and size of grid fields (Giocomo et al., 2007). A different class of models, based on network-level mechanisms rather than intracellular oscillations, has also been put forward to account for grid cell firing (Fuhs and Touretzky, 2006; Kropff and Treves, 2008; McNaughton et al., 2006). These models, at present, have difficulties with capturing some important experimental findings (e.g., phase precession of grid cells), and thus the multiple oscillator theory is still viewed as one of the most viable mechanistic accounts. However, while holding considerable explanatory power, the multiple oscillator theory does not consider realistic membrane potential dynamics. Critically, it relies on the assumption that dendritic independence and democracy can coexist on long timescales in an oscillatory regime. Therefore, understanding the requirements for these two features to emerge in oscillating dendrites would offer important insights into the mechanisms underlying grid field formation, as well as into the nature of ongoing dendritic computations in general.

We begin with a case study of the conflict between maintaining dendritic independence and integrating inputs on long timescales. We demonstrate how grid fields in the multiple oscillator model break down as the independence of dendrites is violated by making realistic assumptions about cellular membrane potential dynamics. We show that the mechanism responsible for the disintegration of grid fields is the phase locking of the dendritic oscillations, which sets an upper limit on the timescale of successful path integration. We then provide a mathematical analysis of the timescale of this phase locking and its dependence on relevant biophysical properties of the dendritic membrane. We find a general trade-off between the speed of phase locking of dendritic oscillators and their ability to influence somatic firing, showing that for ongoing dendritic processing, independence and democracy are essentially incompatible. Finally, we revisit the concrete example of entorhinal stellate cells and show in detailed biophysical simulations that interdendritic coupling dominates, placing these cells at the democratic but nonindependent end of the trade-off.

## Results

### Somato-Dendritic Interactions Disrupt Grid Field Formation in the Multiple Oscillator Model

**Figure 1:**
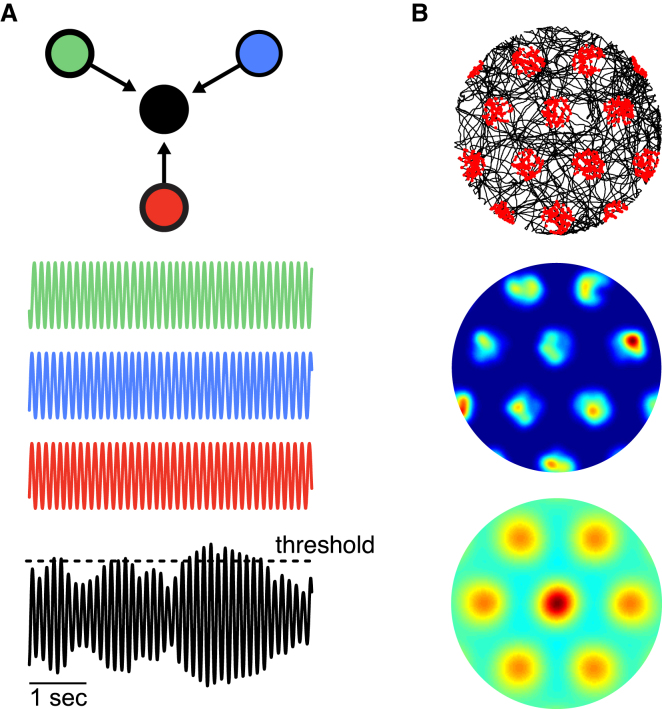
Independent Dendritic Processing of Velocity Signals Yields Stable Grid Fields (A) Structure of the idealized multiple oscillator model. Membrane potentials of the three dendritic oscillators (green, blue, and red) with velocity-modulated frequencies sum at the soma, thereby producing somatic voltage interference patterns (black). Spikes are determined by threshold crossings (dashed line). (B) Grid fields formed by the model. Example of a 10 min simulated trajectory (top panel: black lines) shows that spikes (top panel: red dots) are organized in hexagonally tessellated spatial patterns. Rate map (middle panel) and spatial autocorrelation matrix (bottom panel) show stable grid fields. The rate map color scale is from dark blue (zero) to red (maximum) with a peak rate of 26 Hz. Only the central part of the autocorrelation matrix is shown with color scale from dark blue (*r* = −1) through green (*r* = 0) to red (*r* = 1).

As an emblematic model system to study dendritic computations in a regime where inputs are integrated on long timescales, we implemented the multiple oscillator model of grid cells (Burgess et al., 2007; Hasselmo, 2007; O'Keefe and Burgess, 2005) using three dendritic oscillators (Figure 1A; see also Experimental Procedures). The frequency of each of these oscillators was linearly related to the movement speed of the animal in a particular direction, with the preferred directions of the three oscillators differing by multiples of 120°. Somatic voltage was simply determined by the sum of the dendritic voltages, and spikes were generated when the somatic membrane potential crossed a threshold. As expected, when the dendritic oscillators had different frequencies, the somatic membrane potential showed interference patterns resulting in amplitude variations (black trace in Figure 1A). Using this model, we simulated the activity of a grid cell as an animal randomly explored a circular environment (see Experimental Procedures) and found that it could reproduce the hexagonal-grid-like firing rate fields of entorhinal cells (Figure 1B). Thus, independent dendritic processing of continuous input signals in the idealized multiple oscillator model could produce stable grid field patterns.

**Figure 2:**
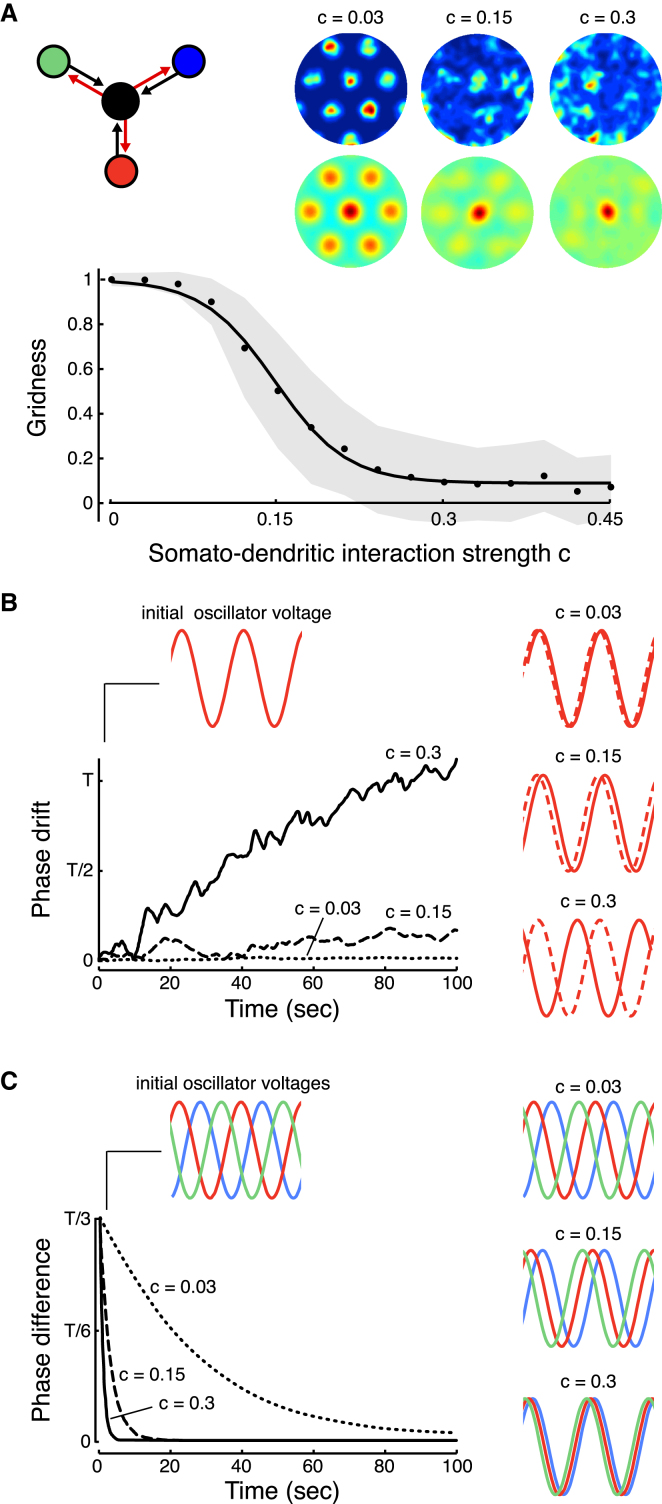
Somato-Dendritic Interactions Disrupt Stable Grid Field Formation due to Dendritic Phase Locking (A) Somato-dendritic coupling (red arrows) progressively disrupts gridness (see Experimental Procedures) with increasing interaction strength *c*. Insets show rate maps (top panels) and spatial autocorrelation matrices (bottom panels) of the activity patterns of a grid cell after a single simulated 5 min exploration with interaction strengths as indicated. Color scales are as in Figure 1, with peak spike rates from left to right as follows: 27 Hz, 35 Hz, and 46 Hz. (B) Grid field disintegration results from imperfect path integration by the oscillators. Interacting oscillator phases drift away from the “correct” values (i.e., phases when oscillators perform perfect path integration with *c =* 0). Phase drifts averaged over the three oscillators shown for *c* = 0.03 (dotted black line), *c* = 0.15 (dashed black line), and *c* = 0.3 (solid black line) are shown. The oscillator period was *T* = 0.125 s. Insets: voltage traces (solid red lines) and “correct” voltage traces (dashed red lines) for a dendritic oscillator at *t* = 0 (left inset) and *t* = 50 s (right insets). (C) Phase drift amplitude and timescale are governed by phase locking of dendritic oscillators: decrease of phase difference between pair of oscillators in absence of external input is shown for *c* = 0.03 (dotted black line), *c* = 0.15 (dashed black line), and *c* = 0.3 (solid black line). The oscillator period was *T* = 0.125 s. Insets show voltage traces of the oscillators (red, blue, and green lines) at *t* = 0 (left inset) and *t* = 5 s (right insets). Oscillators are initialized with phase difference *T*/3.

One of the key assumptions in the multiple oscillator model is that the interaction between the dendritic oscillators and somatic voltage is unidirectional (black arrows in diagram in Figures 1A and 2A): somatic voltage does not affect the oscillators, thus ensuring their perfect independence. However, within a real neuron, electrotonic coupling prevents dendritic compartments from being completely independent. The coupling results from the voltage gradient between the soma and the oscillators, and it is bidirectional: intracellular currents are not only propagated from the dendrites to the soma, but also propagate from the soma back to the dendrites (red arrows in diagram in Figure 2A). When the effects of this coupling were included in the multiple oscillator model (see Experimental Procedures), increasing the strength of coupling caused grid fields to disintegrate (Figure 2A). This disintegration was due to imperfect integration by the oscillators: the phases of the dendritic oscillators gradually drifted away from their ideal values that would have been produced by perfect integration in the original, uncoupled model (Figure 2B). In turn, this drift was caused by a tendency of the oscillators to phase lock with each other. The phase locking of the oscillators was most easily revealed in the absence of external inputs: the oscillator phases drifted toward each other until they became fully synchronized, and full synchronization occurred sooner for stronger coupling (Figure 2C).

Thus, somato-dendritic coupling interferes with the dendritic integration of ongoing inputs, disrupting stable grid field formation by the phase locking of oscillators. The timescale of grid field disintegration is set by the timescale at which dendritic oscillators phase lock, which in turn depends on the strength of coupling between the oscillators. However, the abstract formulation of the multiple oscillator model does not allow a direct assessment of the realistic values of coupling strength, and so it still remains to be determined whether the biophysically realistic timescale of phase locking is sufficient for maintaining stable grid fields. Thus, we formulated a mathematical theory that established a direct relationship between the timescale of phase locking of dendritic oscillations and the biophysical properties of the dendritic membrane.

### A Trade-Off between Dendritic Independence and Democracy for Cable-Coupled Oscillators

**Figure 3:**
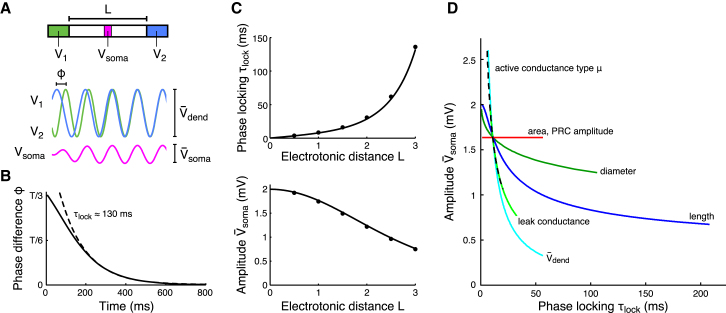
Analysis of Dendritic Oscillator Interactions Shows a Trade-Off between the Speed of Dendritic Phase Locking and Somatic Signal Amplitude (A) Two dendritic oscillators with voltages *V*_1_ (green) and *V*_2_ (blue), oscillation amplitude ${\bar{V}}_{\text{dend}}$, and phase difference *ϕ* are coupled via a cable with electrotonic length *L*. The “somatic” voltage (magenta) at the halfway point of the cable, *V*_soma_, has a maximal oscillation amplitude of ${\bar{V}}_{\text{soma}}$. (B) Coupled dendritic oscillators phase lock and synchronize: in this example, where *L* = 3 and the oscillator period *T* = 125 ms, the phase difference *ϕ* (*t*) approaches 0 with time constant *τ*_lock_ ≈130 ms as indicated by the exponential fit (dashed line). Oscillators are initialized with phase difference *T*/3. (C) Phase locking time constant *τ*_lock_ (top panel) increases, while the somatic oscillation amplitude ${\bar{V}}_{\text{soma}}$ (bottom panel) decreases as a function of *L*. Marks give results from numerical simulations (see Experimental Procedures). (D) Trade-off between phase locking time constant *τ*_lock_ and somatic oscillation amplitude ${\bar{V}}_{\text{soma}}$ persists across biophysical parameter ranges. Curves show the relationship between *τ*_lock_ and ${\bar{V}}_{\text{soma}}$ when each of the parameters indicated is varied (from left to right along the curves) while keeping the other parameters at their standard values (defined in Experimental Procedures): cable length (5–1300 μm), cable diameter (5–0.25 μm), membrane leak conductance (0.05–0.3 mS/cm^2^), active conductance type *μ* (−2.5–2.5), oscillator surface area (50–1200 μm^2^), ${\bar{V}}_{\text{dend}}$ (10–0.4 mV), and PRC amplitude (50–2 ms/mV).

In order to estimate the time constant of phase locking of dendritic oscillators, *τ*_lock_, we mathematically analyzed the phase locking behavior of two sinusoidal oscillators connected by a segment of membrane that itself did not generate intrinsic oscillations (Figure 3A; Experimental Procedures). The most obvious parameter to affect the independence of dendritic oscillators is their electrotonic separation, *L*: the larger it is, the less the oscillators should influence each other. This intuition was confirmed by our analysis (Figure 3B), although *τ*_lock_ still remained in the subsecond range for realistic values of *L* (up to three length constants). Importantly, increasing electrotonic separation also resulted in a decreasing effect of the dendritic oscillators on somatic voltage, ${\bar{V}}_{\text{soma}}$ (Figure 3C). This trade-off between the independence (large phase locking time constant) and democracy (strong signal propagation to the soma) of dendritic oscillations is easily seen when plotting ${\bar{V}}_{\text{soma}}$ against *τ*_lock_ for different lengths of the connecting cable (Figure 3D, blue line).

In order for the dendritic tree to be able to realize more than one functional oscillatory integrator, as also required by the multiple oscillator model of grid cells, democracy and independence must coexist. Thus, we analyzed the effects of various biophysical properties of dendritic membrane potential dynamics on *τ*_lock_ and ${\bar{V}}_{\text{soma}}$ to see what conditions may loosen the democracy-independence trade-off.

Dendritic diameters can vary over a wide range (Stuart et al., 2007), but we found that changing the diameter of the connecting cable did not affect the trade-off substantially: decreasing it increased *τ*_lock_ but also decreased ${\bar{V}}_{\text{soma}}$ at the same time (Figure 3D, dark green line). Increasing the conductance load on the cable (e.g., by shunting inhibition) can also contribute to the electrotonic isolation of parts of the dendritic tree (Bernander et al., 1991; London and Segev, 2001; Rudolph and Destexhe, 2003), but increasing the leak conductance of the connecting cable, to mimic the effects of increased conductance load, also resulted in a similar trade-off (Figure 3D, bright green line). Increasing the amplitude of the dendritic oscillators, ${\bar{V}}_{\text{dend}}$, increased the somatic signal and the coupling currents together; therefore, its effects also followed the general trade-off curve (Figure 3D, cyan line).

Active membrane currents are likely to exist in the nonoscillating parts of the dendritic tree, so we studied the effects of such currents in a way such that the active properties of the connecting cable were summarized by a single parameter, *μ* (see Supplemental Information available online, and also Goldberg et al., 2007; Remme et al., 2009). The sign of *μ* indicated whether the active conductance was regenerative, amplifying perturbations (*μ* < 0, e.g., the persistent sodium current), or restorative, actively counteracting perturbations (*μ* > 0, e.g., the hyperpolarization-activated h-current). We found that changing the active properties of the connecting segment along the regenerative-passive-restorative continuum increased *τ*_lock_ while decreasing ${\bar{V}}_{\text{soma}}$, hence obeying the general trade-off (Figure 3D, black dashed line). In sum, changing most of the relevant parameters characterizing dendritic membrane potential dynamics resulted in a trade-off between dendritic democracy and independence for oscillating dendrites. Moreover, even at the independence end of the trade-off, the phase locking time constant fell far below 1 s for biophysically realizable values of the parameters.

Although the independence-democracy trade-off proved to be robust to changes in most of the properties of the dendrites, we identified one possibility for alleviating it. Making the dendritic oscillators insensitive to inputs by increasing the total amount of current that generated the oscillations naturally made them insensitive to the somato-dendritic currents that caused their coupling. There were two ways in which this could be achieved. First, the surface area of the dendritic oscillators could be increased. Second, the magnitude of the individual currents generating the oscillation could be increased such that the amplitude of the oscillator phase response curve (PRC), describing the amount of phase shift obtained by unit-external perturbations (see Experimental Procedures), was decreased. The effects of these changes were formally equivalent: both increased *τ*_lock_ without affecting ${\bar{V}}_{\text{soma}}$ (Figure 3D, red line). Nevertheless, *τ*_lock_ still remained well below 1 s in a realistic range of these parameters. Further analyses showed that increasing *τ*_lock_ to an extent that allows stable grid fields on a timescale of many minutes would require membrane properties that lie far outside the physiological range (see Supplemental Information). Moreover, an increased insensitivity of the oscillators to the coupling currents also makes them increasingly insensitive to synaptic inputs, because the response to such inputs is also determined by the PRC amplitude and the oscillator surface area (see Supplemental Information and Figure S1, available online).

### Realistic Compartmental Simulations of a Reconstructed Stellate Cell Show Dendritic Phase Locking and Grid Field Failure

**Figure 4:**
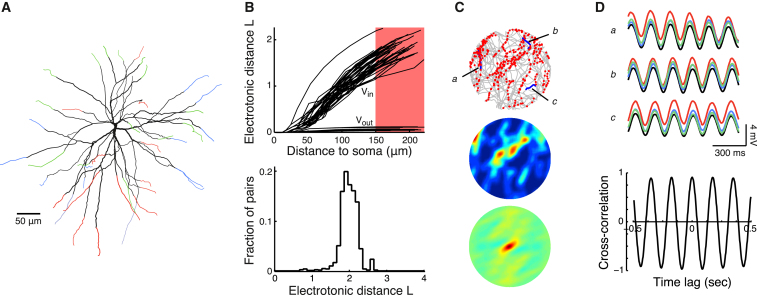
Simulations of Stellate Cell Activity with a Detailed Compartmental Model with Realistic Electrotonic Structure Show Strong Dendritic Coupling (A) Stellate cell model with oscillation-generating conductances in dendritic segments farther than 150 μm from the soma (see Experimental Procedures). The six primary dendrites emerging from the soma are grouped into three clusters, each of which receives external input with a different preferred movement direction (see also Figure 1). Proximal segments (less than 150 μm from soma) of all dendrites are passive. Active segments are color coded according to the cluster to which they belong. (B) The stellate cell is electrotonically compact. Dendro-somatic (*V*_in_) and somato-dendritic (*V*_out_) electrotonic distances (top panel) as well as the electrotonic distances between all pairs of dendritic oscillators (lower panel) fall below three length constants. Red area in top panel denotes active terminal segments of dendrites. (C) Activity of the cell after a simulated 5 min exploration fails to produce stable grid fields. Top panel: trajectory (gray) with threshold crossings (red dots); middle panel: rate map; bottom panel: autocorrelation matrix. Color coding is as in Figure 1 with 8 Hz peak rate. Gridness mean and standard deviation over 10 simulations were 0.10 ± 0.07. (D) Subthreshold membrane potential oscillations are synchronized throughout the stellate cell. Top panel: membrane potentials are plotted for three 1 s trajectories from (C). The colors of the curves each correspond to one dendrite from the three clusters of oscillators in (A). Black trace shows somatic membrane potential. Bottom panel: cross-correlation between the membrane potential of two dendrites from two different dendritic oscillator clusters. See also Figure S4.

Our mathematical theory pointed to a general trade-off between independence and democracy for oscillating dendrites and made specific predictions about how biophysical properties of the dendritic tree influence the phase locking behavior of dendritic oscillators and their effects on somatic voltage. Because the multiple oscillator model suggested that the limiting factor for successful integration by dendritic oscillators is their lack of independence, we constructed a detailed biophysical model of stellate cells, the cell type believed to produce the grid field responses (Figures 4A and S2). In this model we set all parameters to values that allowed the most independence within a realistic range (see Experimental Procedures, Figures S3 and S4, and Table S1).

We began by determining the electrotonic structure of the stellate cell and computing the electrotonic distance between the soma and parts of the dendritic tree along the different dendritic branches, in both the inward dendro-somatic and the outward somato-dendritic directions (Figure 4B, *V*_in_ and *V*_out_, respectively). The maximal electrotonic distance from dendrite to soma was found to be ∼2. The electrotonic distance from soma to dendrites was an order of magnitude smaller, which was due to the tapering of the dendrites with distance from the soma (Carnevale and Johnston, 1982; Rall and Rinzel, 1973; Rinzel and Rall, 1974). Thus, the dendritic voltages were tightly coupled to the somatic voltage and to each other. Our theoretical analysis predicted fast coupling for this range of electrotonic distances (Figure 3C).

We next attempted to generate grid field activity with the compartmental model in the same way as in Figure 1 (see Experimental Procedures). We subdivided the dendritic tree into three groups of dendrites (Figure 4A) with preferred movement directions differing by multiples of 120°. Note that all daughter branches of one dendrite had the same preferred movement direction, thus increasing the total membrane surface area of an oscillator, which should promote independence (see Figure 3D). Despite setting up the simulation in favor of dendritic independence, we found it to be impossible to obtain grid field activity. The oscillating dendritic segments locked so strongly that the cell always showed global synchronized membrane potential oscillations. The rate map of activity as a function of position and its autocorrelation matrix showed a complete absence of the hexagonal grid pattern (Figure 4C). Tracking the membrane potential during three representative 1 s segments of the exploration episode revealed complete synchronization of the soma and the oscillating segments in the dendrites (Figure 4D, top panel). This synchronization was also apparent from the strong cross-correlation between the membrane potential of two dendrites from two different clusters over the complete 5 min exploration episode (Figure 4D, bottom panel).

These results demonstrate that even with optimistic estimates of neuronal properties for independence, the coupling between dendritic segments is too strong to maintain several independent oscillators within one stellate cell. As a consequence, these cells act as single oscillators.

## Discussion

We report mathematical analyses and numerical simulations of interacting dendritic oscillations. Intrinsic subthreshold membrane potential oscillations have been demonstrated in various types of neurons: in stellate cells from entorhinal cortex layer 2 (Alonso and Klink, 1993; Alonso and Llinás, 1989), in neurons from the frontal cortex (Gutfreund et al., 1995), in neurons from the amygdala complex (Pape et al., 1998; Sanhueza and Bacigalupo, 2005), and in pyramidal cells and interneurons from the hippocampal CA1 area (Chapman and Lacaille, 1999; Leung and Yim, 1991). Our results suggest that in such an oscillatory regime, there is a fundamental trade-off between dendritic democracy (Häusser, 2001), expressing how much each oscillator can influence the somatic membrane potential and hence the spiking output of the cell, and dendritic independence (Polsky et al., 2004), or how much each oscillator can integrate its inputs independently of the other oscillators. This is because the same electrotonic coupling that is necessary for dendritic signals to reach the soma also promotes phase locking of dendritic oscillations. Our numerical and analytical results demonstrate that phase locking is essentially unavoidable.

**Figure 5:**
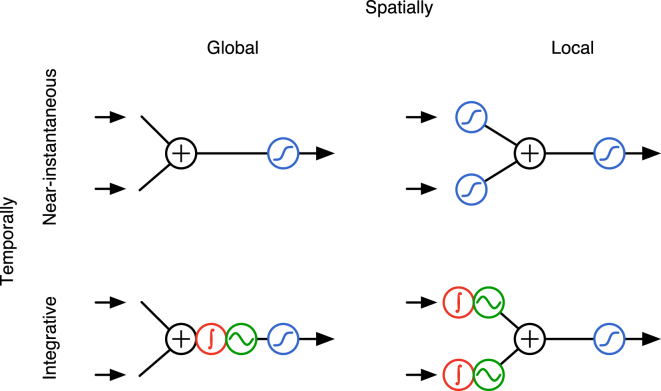
Near-Instantaneous versus Integrative Modes of Dendritic Operation Top panels: during near-instantaneous processing of inputs, the net signal to the soma depends only on the current level of the inputs driving different dendritic subunits. Classical neural network models assumed that dendritic signals are first summed globally and then passed through a nonlinearity (blue) that determines the firing rate of the cell (left, McCulloch and Pitts, 1943). More recent results indicate that dendritic subunits perform local nonlinear operations before their signals are summed at the soma (right, Poirazi et al., 2003). Bottom panels: when inputs are integrated by dendritic oscillations, somatic voltage depends on the history of the inputs. In particular, the information in the inputs is integrated by the oscillation phase. Beyond the timescale on which the dendritic oscillators phase lock, the dendritic tree acts as a single global oscillator integrating all inputs in its phase (red, left). The somatic membrane potential is a (sinusoid-like) nonlinear function of the phase of this global oscillator (green). Below the timescale of phase locking, each dendritic subunit integrates its inputs locally before the dendritic signals are summed at the soma (right).

We find that the time constant of phase locking of dendritic oscillators in biophysically realistic regimes can be on the order of hundreds of milliseconds. This defines two different modes of operation for multiple dendritic oscillations. On timescales shorter than that of phase locking, inputs are integrated in each oscillator locally and independently, and somatic firing is determined by their joint effect. Importantly, this integration can still take place on a timescale that is considerably longer than that suggested simply by membrane time constants. Once phase locking occurs, it causes cells to act as single oscillators. In this mode, synaptic inputs throughout the dendritic tree are integrated in the phase of this single “global” oscillator, which in turn determines somatic firing. Thus, the main difference between local (shorter timescale) and global (longer timescale) dendritic integration of inputs is in the way dendritic nonlinearities and summation act on incoming signals. This difference closely parallels that found between traditional accounts (Hopfield, 1982; McCulloch and Pitts, 1943; Rosenblatt, 1958; Rumelhart and McClelland, 1986) and more recent accounts (Poirazi et al., 2003) of near instantaneous dendritic processing, treating the dendritic tree as a single global computational unit, or as a “network” of multiple local computational units, respectively (Figure 5).

Our results also predict neuronal morphologies that promote independence of dendritic oscillators by slowing down their phase locking. When the oscillation-generating currents are present over a large stretch of dendritic membrane, more current will be needed to shift the phase of this “large” oscillator and, hence, such an oscillator will phase lock more slowly than a “small” oscillator (i.e., one having weak currents or small membrane area). Tufted dendritic terminal branches seem particularly well-suited for creating large but electrotonically separated oscillators. This is because each tuft can contain multiple branches, thus creating an oscillator with a large total surface area, but different tufts can be placed at the ends of different dendrites, thus ensuring the separation of their oscillators. Certain heavily branching cells, such as Purkinje neurons, could exploit such an arrangement to slow down phase locking of dendritic oscillations. Our preliminary calculations estimate that an amount on the order of a hundred tuft-branches is required at the end of each dendrite for the timescale of phase locking to be in the range of tens of seconds. In contrast, dendritic spines cannot be expected to significantly contribute to independence because spines typically increase the dendritic membrane area only by about a factor of 2 (DeFelipe and Fariñas, 1992); thus, *τ*_lock_ remains well below 1 s (see Supplemental Information).

The electrotonic structure of cortical stellate cells—the cell type thought to produce the grid field activity—is quite unlike that predicted to be ideal for dendritic independence. This is because the stellate cell is electrotonically compact, having four to six primary dendrites that do not branch extensively (Klink and Alonso, 1997), and tapering of the dendrites further supports strong coupling of dendritic voltages to the soma (Carnevale and Johnston, 1982; Rall and Rinzel, 1973; Rinzel and Rall, 1974). Although inhibitory inputs impinging on the dendrite can increase the effective membrane conductance through their shunting effects, our results show that the slowing down of phase locking brought about by such shunting is limited (Figure 3D). Indeed, in a compartmental model of a spiny stellate cell, we found that with realistic biophysical properties the interdendritic coupling was so strong that rather than supporting independent dendritic oscillations, the cell acted as a single oscillator (Figure 4), even when strong shunting effects were taken into account (Figures S4B, S4D, and S4E). These results suggest that at least certain network mechanisms need to be taken into account for explaining the emergence of grid fields. Such mechanisms for generating grid fields have been proposed previously (Fuhs and Touretzky, 2006; Kropff and Treves, 2008; McNaughton et al., 2006). However, while network models can explain some properties of grid cell activity that the multiple oscillator framework cannot, such as the correlations between grid cells possessing similar spatial periods (Fyhn et al., 2007), presently they do not account for some other important data that are naturally captured by oscillation-based theories, such as phase precession in grid cells (Hafting et al., 2008). Therefore our analysis calls for continuing investigations into the biophysical bases of grid cell firing by ruling out a candidate mechanism that otherwise seems deceptively fit for explaining a wide array of data.

The dendritic democracy-independence trade-off we identified is unique to ongoing dendritic processing, such as that achieved by oscillations: if inputs are integrated only on short timescales, then the dendritic processing is essentially over by the time different dendritic branches would start interacting. Previous studies of dendritic integration focused on such near-instantaneous transformations of dendritic inputs into somatic outputs and thus did not address this issue (Poirazi et al., 2003). More generally, the contributions of active ionic conductances to dendritic processing have almost exclusively been studied in the context of near-instantaneous processing (Gasparini et al., 2004; Larkum et al., 2009; Losonczy and Magee, 2006; Nevian et al., 2007; Wei et al., 2001). The analysis presented here represents the first step toward understanding dendritic computation in another and hitherto scarcely studied dynamical regime, that of ongoing dendritic oscillations, in which active processes may play a crucial role, and identifies computationally relevant features that are unique to this mode of operation.

## Experimental Procedures

### Multiple Oscillator Model

The single-cell mechanism to generate grid fields described by Burgess et al. (2007) relies on the interference pattern that emerges at the soma from distinct independent oscillators in the cell. We considered a minimal model consisting of three sinusoidal dendritic oscillators connected to the soma (see Figure 1A). The frequency of dendritic oscillator *i* was modulated by external input *I*_vel,*i*_(*t*) = *β s*(*t*) cos(*ψ*(*t*) − *ψ_i_*) that signaled the animal's speed *s*(*t*) (in cm/s) and direction of motion *ψ*(*t*), using scaling constant *β* = 2.9 ·10^−3^ and setting the preferred direction *ψ_i_* to a multiple of 120°. To account for somato-dendritic coupling, each oscillator also received a current *I*_sd,*i*_(*t*) = *c*(*V*_soma_(*t*) − cos *ϑ_i_*), resulting from the difference of its voltage and the somatic voltage *V*_soma_(*t*), with parameter *c* controlling the somato-dendritic interaction strength. Hence, the phase *ϑ_i_* of oscillator *i* evolved as

(1) $$\frac{d\vartheta_{i}}{dt}=\omega +Z\left(\vartheta_{i}\right)\left(I_{\text{vel},i}\left(t\right)+I_{\text{sd},i}\left(t\right)\right)$$

with oscillator frequency *ω* = 2π/*T* and period *T* = 0.125 s, and where *Z*(*ϑ_i_*) = *ω*(1 − *ɛ*(1 + sin *ϑ_i_*)) determined the change of the oscillator frequency in response to input arriving at phase *ϑ_i_* with *ɛ* = 0.05. Note that the simulation shown in Figure 1 used *c* = 0. Somatic voltage was simply determined by the average of the dendritic voltages:

(2) $$V_{\text{soma}}\left(t\right)=\frac{1}{3}\left(\cos\vartheta_{1}+\cos\vartheta_{2}+\cos\vartheta_{3}\right)$$

Spike output resulted when the somatic membrane potential crossed a threshold *V*_thresh_ = 0.7.

### Random Exploration of an Environment

Simulations in Figures 1, 2, and 4 used random explorations, which were generated as in Hasselmo et al. (2007). The algorithm involved random steps and a momentum term leading to smooth changes in direction and speed, resembling the exploratory behavior of rats. The changes in spatial location Δ*x* and Δ*y* (in centimeters) at time *t* (in seconds) were determined by difference equations:

(3) $$\begin{array}{l} \Delta x\left(t+\Delta t\right)=S\left(1-m\right)p_{x}+m\Delta x\left(t\right)\\ \Delta y\left(t+\Delta t\right)=S\left(1-m\right)p_{y}+m\Delta y\left(t\right) \end{array}$$

where Δ*t* = 0.005 s, *S* = 1.25 cm, inertia *m* = 0.9975, and *p_x_* and *p_y_* were independent random variables from a standard normal distribution sampled independently in each time step of the simulation. The environment was circular with radius 50 cm. If the random movement crossed the barrier in direction *x*, the movement for that time step was reversed Δ*x*(*t*) = −*R* Δ*x*(*t*), with *R* = 0.5, and similarly for direction *y*. Speed and direction from the above algorithm were calculated as $s\left(t\right)=\sqrt{\Delta x^{2}+\Delta y^{2}}/\Delta t$ and *ψ*(*t*) = arctan(Δ*y*(*t*)/Δ*x*(*t*)).

The smoothed rate maps, spatial autocorrelation matrices, and gridness measure of the model output were calculated as in Sargolini et al. (2006). Gridness mean and standard deviation in Figure 2 was determined for 100 repetitions of 5 min explorations for each interaction strength value *c*. The gridness measure was then normalized by the mean value observed with *c* = 0.

### Mathematical Analysis

To determine the dependence of the phase locking time constant *τ*_lock_ and somatic oscillation amplitude ${\bar{V}}_{\text{soma}}$ on dendrite and oscillator parameters, we analyzed a system of two oscillators that are coupled via a cable of length *l* cm (see Figure 3A), building on previous work analyzing the steady-state phase-locked configuration of dendritically coupled oscillators (Remme et al., 2009). The full analysis can be found in the Supplemental Information. What follows is a summary of the methods and results. The membrane potential *V_i_*(*t*) of each dendritic oscillator is described by a sinusoidal function with amplitude ${\bar{V}}_{\text{dend}}$ and angular frequency *ω*. The dynamics of the cable connecting the oscillators are determined by the cable equation with membrane time constant *τ* and length constant *λ*, giving the cable an electrotonic length *L* = *l*/*λ*. The cable also expressed voltage-dependent conductances modeled with a single gating variable *m*(*x*,*t*).

The oscillators interacted via the currents flowing along the cable. We considered that the perturbations via the cable only affect the oscillator phases. The change of an oscillator's phase in response to an infinitesimally small and short perturbation at a particular phase is described by its infinitesimal PRC (Izhikevich, 2007). We considered a sinusoidal PRC with amplitude *Q*. To determine the perturbations to the oscillators, we needed to solve the cable equation with the oscillators at the ends of the cable giving the periodically forced end conditions of the cable. After linearizing the cable equation, we obtained equations describing the evolution of the phase difference between the oscillators from which we derived the phase locking time constant

(4) $$\tau_{\text{lock}}=\frac{a\tau }{\pi d\lambda \omega Q{\bar{V}}_{\text{dend}}}\frac{1}{\rho \left|\cos\xi \right|}$$

with oscillator surface area *a* and cable diameter *d*, and where |cos *ξ*| is the absolute value of cos *ξ*, and *ρ* and *ξ* denote the signal attenuation and phase shift resulting from cable filtering. Note that *τ*, *λ*, *ρ*, and *ξ* are functions of the cable parameters: membrane leak conductance *g*_L_, intracellular resistivity *R*_i_, membrane capacitance *C*_m_, length *l*, diameter *d*, and the active current's density, time constant, and type *μ* (see Supplemental Information). The sign of *μ* indicates whether the active conductance is regenerative, amplifying perturbations (*μ* < 0, e.g., the persistent sodium current), or restorative, actively counteracting perturbations (*μ* > 0, e.g., the hyperpolarization-activated h-current).

Considering the voltage at the middle of the cable to be the “somatic” voltage *V*_soma_(*t*), we can write the maximal somatic oscillation amplitude as

(5) $${\bar{V}}_{\text{soma}}=\frac{{\bar{V}}_{\text{dend}}}{\left|\cosh\left(bL/2\right)\right|}$$

where complex number *b* describes the cable filtering and is a function of the cable parameters.

Standard parameter values used in Figure 3 for the oscillators were: ${\bar{V}}_{\text{dend}}=2$ mV, PRC amplitude *Q* = 10 ms/mV, and oscillator area *a* = 236 μm^2^; and for the cable, parameter values were: length *l* = 500 μm, diameter *d* = 0.6 μm, membrane conductance *g*_L_ = 0.05 mS/cm^2^, membrane capacitance *C*_m_ = 1 μF/cm^2^, and intracellular resistivity *R*_i_ = 200 Ω cm. Active current parameters when *μ* was varied were: activation time constant *τ*_m_ = 1 ms and relative density *γ*_R_ = 1.25. Otherwise the cable was passive, i.e., *μ* = 0 and *γ*_R_ = 1.

### Cable Model with Two Weakly Coupled Oscillators

Numerical simulations for Figure 3 used Andronov-Hopf oscillators, written in complex form as

(6) $$\frac{dz_{i}}{dt}=\left({\bar{V}}_{\text{dend}}^{2}+i\omega \right)z_{i}-{\left|z_{i}\right|}^{2}z_{i}+ɛp_{i}\left(t\right)$$

such that oscillator voltage *V_i_*(*t*) is equal to the real part of *z_i_*(*t*) and has amplitude ${\bar{V}}_{\text{dend}}=2$ mV. The cable was discretized into isopotential compartments with length Δ*x* ≤ 0.05 *λ*. The perturbations from the cable to oscillator *i* were computed as $ɛp_{i}\left(t\right)=ɛ\left(U_{1}\left(t\right)-V_{i}\left(t\right)\right)/\Delta x$ with *U*_1_(*t*) denoting the membrane potential of the first cable compartment connecting to oscillator *i*, and parameter *ɛ* = 0.006 cm/ms describing the coupling between cable and oscillators. Cable parameters were set to standard values as indicated above.

### Compartmental Model of a Stellate Cell with Multiple Oscillators

Numerical simulations for Figure 4C used a compartmental model of a reconstructed cortical spiny stellate cell with representative morphology (Klink and Alonso, 1997). Dendritic segments farther than 150 μm from the soma had oscillation-generating conductances (see below). This distance from the soma to the oscillators was set to be optimal for making phase locking as slow as possible and was computed using the above mathematical framework (see Figure S3). The soma and dendritic segments less than 150 μm from the soma were passive with membrane conductance *g*_L_ = 0.05 mS/cm^2^ and reversal potential *E*_L_ = −80 mV. The model used intracellular resistivity *R*_i_ = 200 Ω cm and membrane capacitance *C*_m_ = 1 μF/cm^2^. Dendrites were discretized into compartments with length Δ*x* ≤ 0.05 *λ*. Oscillations were induced with uniform current injection *I* = 0.3 μA/cm^2^ at both the active and passive segments. The electrotonic distance was determined for 8 Hz voltage oscillations at the mean somatic voltage of −51.5 mV. Simulations of this model and calculation of the electrotonic distances were carried out with NEURON (Hines and Carnevale, 1997).

During random exploration simulations the active segments received velocity-dependent external input *I*_vel_(*t*) generated with the algorithm described above. The six primary dendrites emerging from the soma were grouped into three clusters with the preferred velocity direction *ψ_i_* of the clusters differing by multiples of 120°. The amplitude of the external input *I*_vel_(*t*) was scaled by a factor *K*_input_ = 0.005 (i.e., *β* → *K*_input_ *β*) to adjust for the model's input resistance. Spike times were generated by crossings of a threshold at −49.7 mV. For additional simulations using different membrane parameters see Figure S4 and Table S1.

### Oscillator Model

Subthreshold oscillations in the active segments of the spiny stellate cell model were generated by an interaction between a persistent sodium current, *I*_NaP_, and a hyperpolarization-activated cation current, *I*_h_ (Dickson et al., 2000). Fransén et al. (2004) previously reported a detailed biophysical model capable of producing such oscillations. However, in that model, subthreshold oscillations with appropriate characteristics for entorhinal stellate cells (with realistic voltage amplitudes and frequencies) were restricted to a very limited set of parameter values (see also White et al., 1995), making explorations of parameters necessary for our study unfeasible. Moreover, we also observed significant, hard-to-track dependencies between the range of parameters suitable for subthreshold oscillations and other dendritic parameters we varied in this study. We thus chose to resolve these technical problems, following White et al. (1995), by systematically reducing the Fransén et al. (2004) model to a two-variable oscillator model (see Supplemental Information). When reducing the model, care was taken to ensure that the resulting reduced oscillator model preserved relevant dynamical properties—such as the voltage trajectory and the PRC—of the full oscillator model (Figure S2). The oscillator voltage (in mV) and *I*_h_ gating variable *r*(*V*,*t*) evolved according to:

(7) $$\begin{array}{c} C_{\text{m}}\frac{dV}{dt}=-g_{\text{L}}\left(V-E_{\text{L}}\right)-g_{\text{h}}r\left(V-E_{\text{h}}\right)-g_{\text{NaP}}n_{\infty }\left(V\right)\left(V-E_{\text{Na}}\right)+I\\ \frac{dr}{dt}=\varphi \frac{r_{\infty }\left(V\right)-r}{\tau_{\text{r}}\left(V\right)} \end{array}$$

with *C*_m_ = 1 μF/cm^2^, *g*_L_ = 0.08 mS/cm^2^, *E*_L_ = −84 mV, *g*_h_ = 3.1 mS/cm^2^, *E*_h_ = −20 mV, *g*_NaP_ = 0.094 mS/cm^2^, *E*_Na_ = 48 mV, and *φ* = 0.014, and where $n_{\infty }\left(V\right)=1/2\left[1+\tanh\left(\left(V+48.7\right)/8.8\right)\right]$, $r_{\infty }\left(V\right)=1/2\left[1+\tanh\left(\left(V+74.2\right)/-14.4\right)\right]$, and $\tau_{\text{r}}\left(V\right)=1/\cosh(\left(V+74.2\right)/-28.8)$.

## Supplemental Information

Document S1. Supplemental Figures, Table, and Experimental Procedures
