# Supplementary material for: Democracy-Independence Trade-Off in Oscillating Dendrites and Its Implications for Grid Cells
Source: Neuron. 2010 May 13;66(3):429–37. doi: 10.1016/j.neuron.2010.04.027 (PMC3501565; doi:10.1016/j.neuron.2010.04.027)
Supplement: Document S1. Supplemental Figures, Table, and Experimental Procedures [file mmc1.pdf]

Neuron, volume *66*

Supplemental Information

Democracy-Independence Trade-Off in Oscillating Dendrites and Its Implications for Grid Cells

Michiel W. H. Remme,[1,]* Máté Lengyel,[2] and Boris S. Gutkin[1]

[1]Group for Neural Theory, Département d'Études Cognitives, École Normale Supérieure, 29 rue d'Ulm, 75005 Paris, France

[2]Computational and Biological Learning Lab, Department of Engineering, University of Cambridge, Trumpington Street, Cambridge CB2 1PZ, UK

*Correspondence: michiel.remme@nyu.edu

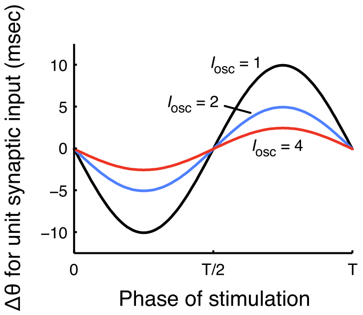

Figure S1. Increasing the magnitude of oscillation-generating currents decreases the response to synaptic input (related to Figure 3). Curves show the oscillator phase shift in response to a 1 mV synaptic input for 3 magnitudes of total oscillator current, denoted by scaling factor "I_{osc}". The magnitude of the oscillation-generating currents can be increased by increasing area *a* of the oscillating compartment and/or by increasing the conductance densities. Parameter *a* is included in ε (see Equation S13) and varying the conductance densities scales the PRC amplitude *Q* (see for example Izhikevich, 2007, chapter 10). The change of the oscillator phase shift $\Delta\theta$ in response to synaptic input is determined by the product of the input, a parameter ε, and the phase response curve, similar to the oscillator response to interdendritic currents (see equation S2), except that ε depends on the synaptic conductance rather than on the intracellular resistance (see equation S13).

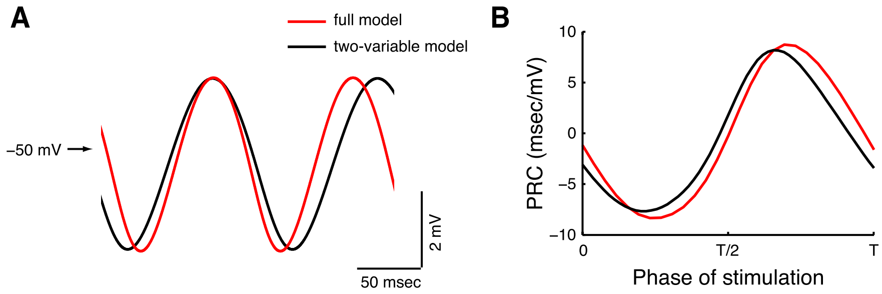

Figure S2. Full and reduced model of stellate cell subthreshold oscillations show similar voltage trajectory and phase response curve (related to Figure 4 and Supplemental Figure S4). Panels show the voltage trajectory (A) and phase response curve (B) of a dendritic compartment with oscillation-generating currents from Fransén et al. (2004) ("full model", red lines) and from the reduced model described in Experimental Procedures ("two-variable model", black lines). Parameters of both models were adjusted to generate ongoing oscillations with frequency ~ 8 Hz and a mean membrane potential of ~ –50 mV. Adjusted parameters of the two-variable model were $g_L = 0.149$ mS/cm^2, $g_h = 1.35$ mS/cm^2, $g_{NaP} = 0.044$ mS/cm^2, $E_L = -78$ mV, and $I = 0.9$ µA/cm^2. The full model is described in Fransén et al. (2004), using $g_L = 0.156$ mS/cm^2, $g_{h(fast)} = 1.47$ mS/cm^2, $g_{NaP} = 0.087$ mS/cm^2, $E_L = -78$ mV, $E_{Na} = 48$ mV, and $I = 0.9$ µA/cm^2.

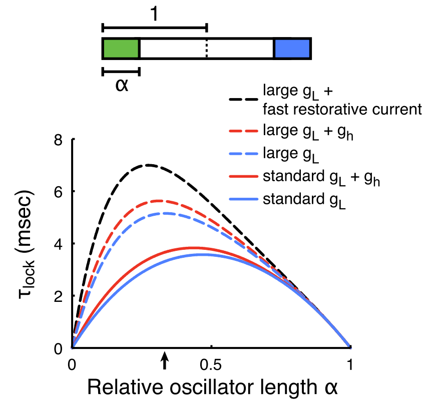

Figure S3. Optimal dendritic conductances and oscillator size to maximize the phase locking time constant (related to Figure 4 and Supplemental Figure S4). To determine the optimal distance in the stellate cell model between the oscillators and the soma (see Figure 4 and Supplemental Figure S4) we used our analytical framework to compute the phase locking time constant τ_{lock} for a cable with a fixed total length l_{total} of which the oscillators take up a variable fraction α. We consider that the diameter for the oscillating segments and the connecting cable is equal, hence the relative length of one oscillator is $\alpha\, l_{total}/2$. Using Equation S14 we computed τ_{lock} as a function of α for a passive cable with standard conductance load ("standard g_L", solid blue line) or high conductance load ("large g_L", dashed blue line), and for those two cables with an additional h-type, restorative conductance ("standard $g_L + g_h$", solid red line, and "large $g_L + g_h$", dashed red line). In order to obtain the maximal τ_{lock}, we also considered a cable with a high conductance load and a hypothetical restorative current that activates very quickly ("large g_L + fast restorative current", dashed black line). Note that the active cable current in Figure 3D used the same fast activation. Standard and high conductance load cables used $g_L = 0.05$ mS/cm^2 and $g_L = 0.3$ mS/cm^2, respectively. The restorative h-type current was based on the I_h that was included in the stellate cell simulations shown in Supplemental Figure S4C-E. Active current parameters were computed at $V = -50$ mV using $g_h = 1.1$ mS/cm^2 and yielded $\mu = 2.9$ with relative density $\gamma_R = 1.7$ for the cable with standard conductance load, and $\mu = 0.5$ with relative density $\gamma_R = 1.1$ for the cable with large conductance load. Activation time constant was $\tau_m = 50$ msec for the h-type restorative current and $\tau_m = 1$ msec for the hypothetical fast restorative current. The total cable length including the two oscillators was $l_{total} = 450$ µm, diameter $d = 0.6$ µm, membrane capacitance $C_m = 1$ µF/cm^2, and intracellular resistivity $R_i = 200$ Ω cm. Oscillator voltage amplitude was $\bar{V}_{dend} = 2$ mV and PRC amplitude was $Q = 10$ ms/mV. Note that most stellate cell model simulations used $\alpha \approx 75$ µm / 225 µm = 0.33 (black arrow).

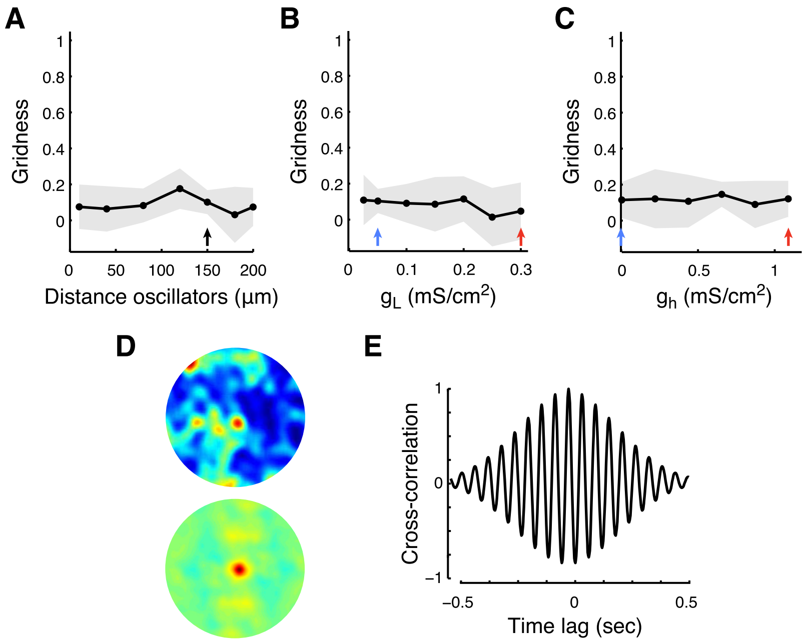

Figure S4. Simulations of stellate cell activity (related to Figure 4). Activity of the stellate cell after simulated explorations failed to produce grid-fields over a wide range of model parameters. Panels A-B-C show gridness mean and standard deviation for 10 explorations of 5 minutes. A: Distance between oscillators and soma was increased from 10 to 200 µm (see Experimental Procedures and Figure 4B) thereby also decreasing the oscillator surface area. Dendrites and soma connecting the oscillators are passive. Parameters are defined by Models 1–7 in Supplemental Table S1. Arrow indicates value used in all other simulations. B: Conductance load on dendrites connecting oscillators was varied by increasing the leak conductance g_L from 0.025 to 0.3 mS/cm^2. Parameters are defined by Models 3 and 8–13 in Supplemental Table S1. Blue and red arrows indicate values used in Figure 4 and panels D-E, respectively. C: A restorative current was added to the soma and dendrites connecting the oscillators. For this we used the same hyperpolarization-activated cation current I_h as for the active segments (see Experimental Procedures), varying its density g_h from 0 to 1.1 mS/cm^2. This range corresponds to varying the parameter μ in our mathematical analysis from 0 to 2.9 (see Figure 3D). Parameters are defined by Models 3 and 14–18 in Supplemental Table S1. Blue and red arrows indicate values used in Figure 4 and panels D-E, respectively. D-E: Using parameters that allowed for the most independence between dendritic oscillators, the activity of the stellate cell still failed to produce stable grid-fields and showed strong phase locking throughout the dendritic tree. Our mathematical analysis predicted that adding a large conductance load and restorative currents to the dendritic segments increases τ_{lock} (see Figure 3D and Supplemental Figure S3). We used the maximal conductance densities shown in panels B and C (red arrows). We also used a distance between the oscillators and soma that was close to optimal for slowing down phase locking (see Supplemental Figure S3 and arrow in panel A). Parameters are defined by Model 19 in Supplemental Table S1. Gridness mean and standard deviation for 10 simulated explorations of 5 minutes was 0.09 ± 0.12. Rate map (D, top panel) and autocorrelation matrix (D, bottom panel) of a single simulation illustrates absence of grid pattern (color coding as in Figure 1 with peak rate 15 Hz). Membrane potentials were strongly correlated between dendrites from different oscillator clusters (E). Note that the large membrane conductances decreased the oscillation period to $T = 56$ msec.

Model	Dendrites g_L	Dendrites E_L	Dendrites g_h	Oscillator g_L	Oscillator E_L	Oscillator g_h	Oscillator g_{NaP}	D_{osc}	K_{input}	V_{th}
1	0.050	−84	0	0.080	−84	15.500	0.470	200	0.0050	−58.30
2	0.050	−82	0	0.080	−84	8.060	0.244	180	0.0050	−51.25
3	0.050	−80	0	0.080	−84	3.100	0.094	150	0.0050	−49.70
4	0.050	−80	0	0.080	−84	1.860	0.056	120	0.0050	−47.75
5	0.050	−76	0	0.080	−84	1.240	0.038	80	0.0050	−48.90
6	0.050	−68	0	0.080	−84	1.023	0.031	40	0.0050	−48.50
7	0.050	−60	0	0.080	−84	0.976	0.030	10	0.0050	−49.00
8	0.025	−89	0	0.080	−88	2.387	0.072	150	0.0040	−48.00
9	0.100	−76.5	0	0.080	−84	4.960	0.150	150	0.0056	−49.70
10	0.150	−76.5	0	0.080	−84	6.200	0.197	150	0.0059	−52.25
11	0.200	−78	0	0.080	−84	7.750	0.244	150	0.0063	−55.00
12	0.250	−79	0	0.080	−84	8.680	0.273	150	0.0071	−58.13
13	0.300	−82	0	0.080	−84	9.920	0.320	150	0.0083	−60.80
14	0.050	−86	0.217	0.080	−84	3.410	0.103	150	0.0056	−48.03
15	0.050	−93.5	0.434	0.080	−84	3.720	0.113	150	0.0059	−47.75
16	0.050	−101	0.651	0.080	−84	4.030	0.122	150	0.0063	−47.87
17	0.050	−110	0.868	0.080	−84	4.340	0.132	150	0.0067	−47.74
18	0.050	−118	1.085	0.080	−84	4.650	0.141	150	0.0067	−48.13
19	0.300	−90	1.085	0.100	−90	10.850	0.329	150	0.0083	−58.50

Table S1. Model parameters for simulations of stellate cell activity (related to Figure 4 and Supplemental Figure S4). From left to right, columns give current parameters in soma and dendrites connecting the oscillators ("Dendrites"), oscillator current parameters ("Oscillator"), distance between soma and oscillators ("D_{osc}"), input scaling factor ("K_{input}"), and somatic spiking threshold ("V_{th}"), with conductances in mS/cm^2, E_L and V_{th} in mV, and D_{osc} in µm. Remaining model parameters are described in Experimental Procedures. Simulations in Figure 4 of main text use Model 3 (blue shading). Simulations in Supplemental Figure S4D-E use Model 19 (red shading). Varying parameters of the soma and dendritic segments required adjustments of the oscillator parameters for the model to generate membrane potential oscillations. Note that certain parameter settings require strongly hyperpolarized leak reversal potentials E_L to counteract the depolarizing effect of g_h and g_{NaP} and maintain the subthreshold oscillations. Furthermore, the amplitude β of the external input $I_{vel,i}(t)$ was scaled by a factor K_{input} (see Experimental Procedures) to adjust for the model's input resistance. The somatic spiking threshold V_{th} was set such that the model produced mean firing rates of ~ 2 Hz.

Supplemental Experimental Procedures: Derivation of the phase locking time constant

We performed a mathematical analysis to determine how fast phase locking of dendritic oscillators occurs as a function of the oscillator properties and the properties of the membrane segment connecting the oscillators. Consider a system of two identical oscillators with natural frequency f (in Hz) that are coupled via a cable of length l cm, with oscillator $i = 1,2$ located at $x = 0$ and $x = l$, respectively (see Figure 1A). The membrane potential $V_i(t)$ (in millivolts) of each dendritic oscillator is described by a sinusoidal function:

$$V_i(t) = \bar{V}_{\mathrm{dend}} \cos(\omega(t + \theta_i)) + V_{\mathrm{R}} \tag{S1}$$

with dendritic oscillator amplitude $\bar{V}_{\mathrm{dend}}$ (in millivolts), angular frequency $\omega = 2\phi f$, phase shift θ_i, and resting membrane potential V_{R}. We consider the oscillators are weakly coupled (i.e. the interactions only affect the oscillators' phases). We can then write the changes in the phase shifts of the oscillators as

$$\theta_i = \varepsilon Z(t) p_i(t) \tag{S2}$$

where the positive parameter $\varepsilon << 1$, and $Z(t)$ is the infinitesimal phase response curve (PRC) which is assumed to be identical for both oscillators. It describes the change of the oscillator's phase shift in response to an infinitesimally small and short perturbation at a particular phase (Izhikevich, 2007). Here we consider $Z(t) = -Q \sin(\omega t)$, where Q is the amplitude of the PRC (in seconds per millivolt). Note that when $Q = 1/\omega\bar{V}_{\mathrm{dend}}$ we obtain the PRC of Andronov-Hopf oscillators, the minimal dynamical system to produce the sinusoidal limit cycle oscillations in equation S1. The perturbations $p_i(t)$ result from the axial currents that flow between the cable and oscillator i. The passive properties of the cable are determined by a membrane time constant τ (in seconds), a leak reversal potential E_{L} (in millivolts), and a length constant λ (in centimeters), giving the cable an electrotonic length $L = l/\lambda$. The cable also expresses a voltage-dependent conductance with reversal potential E_{m}. The dynamics of this conductance are determined by a single gating variable $m(x,t)$ with activation function $m_\infty(V)$ and time constant τ_{m}. The equations governing the membrane potential $V(x,t)$ and the gating variable $m(x,t)$ along the cable are

$$\begin{aligned} \tau \frac{\partial}{\partial t} V(x,t) &= \lambda^2 \frac{\partial^2}{\partial x^2} V(x,t) - (V(x,t) - E_{\mathrm{L}}) - \gamma_{\mathrm{m}} m(x,t)(V(x,t) - E_{\mathrm{m}}) \\ \tau_{\mathrm{m}} \frac{\partial}{\partial t} m(x,t) &= m_{\square}(V(x,t)) - m(x,t) \end{aligned} \tag{S3}$$

where γ_{m} is the ratio of the maximal conductance of the active current to the passive membrane conductance. In order to determine the perturbations to the oscillators we need to solve equation S3 with the oscillators at the ends of the cable giving the periodically forced end conditions of the cable. For this, we first linearize equation S3 about the membrane voltage V_{R} around which the membrane potential oscillates, leading to the quasi-active approximation for the cable (Sabah and Leibovic, 1969; Koch, 1984). We define $U(x,t)$ as the difference between the oscillating solution and the resting membrane potential V_{R}, i.e. $U(x,t) \equiv V(x,t) - V_{\mathrm{R}}$ and we define $w(x,t)$ analogously as $w(x,t) \equiv m(x,t) - m_\infty(V_{\mathrm{R}})$. The equations describing the quasi-active cable read

$$\begin{aligned} \tau \frac{\partial}{\partial t} U(x,t) &= \lambda^2 \frac{\partial^2}{\partial x^2} U(x,t) - \gamma_{\mathrm{R}} U(x,t) - \gamma_{\mathrm{m}} (V_{\mathrm{R}} - E_{\mathrm{m}}) w(x,t) \\ \tau_{\mathrm{m}} \frac{\partial}{\partial t} w(x,t) &= \frac{\partial}{\partial V} m_\infty(V_{\mathrm{R}}) U(x,t) - w(x,t) \end{aligned} \tag{S4}$$

where $\gamma_R = 1 + \gamma_m m_\infty(V_R)$. Using the oscillators from equation S1 as the periodically forced end conditions for equation S4 we can write the solution as

$$U(x,t) = \bar{V}_{\mathrm{dend}} \operatorname{Re}\left[\frac{\sinh\left(b\left(L - x/\lambda\right)\right)}{\sinh(bL)} e^{i\omega(t+\theta_1)} + \frac{\sinh\left(b\,x/\lambda\right)}{\sinh(bL)} e^{i\omega(t+\theta_2)}\right] \tag{S5}$$

where Re[z] is the real part of the complex number z and where

$$b = \sqrt{\gamma_R + \frac{\mu}{1+(\omega\tau_m)^2} + i\omega\left(\tau - \frac{\mu\tau_m}{1+(\omega\tau_m)^2}\right)} \tag{S6}$$

with $\mu = \gamma_m (V_R - E_m)\frac{\partial}{\partial V} m_\infty(V_R)$. We can then show that the perturbation to oscillator $i = 1$ reads

$$p_1(t) = \frac{\partial}{\partial x} U(0,t) = \frac{\bar{V}_{\mathrm{dend}}}{\lambda} \operatorname{Re}\left[\frac{b}{\sinh(bL)} e^{i\omega(t+\theta_2)} - \coth(bL) e^{i\omega(t+\theta_1)}\right] \tag{S7}$$

We want to describe the evolution of the phase difference $\phi(t) = \theta_2(t) - \theta_1(t)$. For this we first need to determine the phase interaction function $H_i(\phi)$ that describes the average effect of perturbation $p_i(t)$ on the phase of oscillator i over a cycle of period $T = 2\pi/\omega$. For oscillator $i = 1$ this interaction function reads

$$\begin{aligned} H_1(\phi) &= \frac{1}{T}\int_0^T Z(t) p_1(t+\phi)\,dt \\ &= \frac{Q\bar{V}_{\mathrm{dend}}}{2\lambda} \rho \sin(\omega\phi + \xi) + \nu \end{aligned} \tag{S8}$$

where

$$\rho = \left|\frac{b}{\sinh(bL)}\right| \tag{S9}$$

$$\xi = \arg\left[\frac{b}{\sinh(bL)}\right] \tag{S10}$$

and ν is a constant, and where |z| and arg[z] are the absolute value and the angle of the complex number z, respectively. The interaction function $H_2(\phi)$ can be determined similarly. We now have the equation describing the evolution of the phase difference between the two oscillators:

$$\dot{\phi} = -\varepsilon \frac{Q\bar{V}_{\mathrm{dend}}}{\lambda} \rho \cos(\xi) \sin(\omega\phi) \tag{S11}$$

The fixed points of this differential equation, i.e. the points where $\dot{\phi} = 0$, are $\phi = k\,\pi/\omega$ where k is an integer. The stable fixed points are those points where $d\dot{\phi}/d\phi < 0$. Close to the stable fixed points the term $\sin(\omega\phi)$ is approximately linear and the phase locking time constant is

$$\tau_{\mathrm{lock}} = \frac{\lambda}{\varepsilon\omega Q\bar{V}_{\mathrm{dend}}} \frac{1}{\rho\left|\cos\xi\right|} \tag{S12}$$

The parameter ε can be described in terms of cable and oscillator parameters:

$$\varepsilon = \frac{\pi d^2}{4R_i C_m a} = \frac{\pi d \lambda^2}{a\tau} \tag{S13}$$

where R_i is the intracellular resistivity (in Ωcm), a is the surface area of one oscillator (in cm^2) and C_m is the specific membrane capacitance (in µF/cm^2). The second equality uses $\lambda = \sqrt{d/4R_i g_L}$ and $\tau = C_m / g_L$, where g_L is the specific membrane conductance (in S/cm^2). Hence we can write the phase locking time constant as

$$\tau_{\text{lock}} = \frac{a\,\tau}{\pi\, d\, \lambda\, \omega\, Q\, \bar{V}_{\text{dend}}} \frac{1}{\rho \left|\cos \xi\right|} \quad \text{(S14)}$$

Amplitude of the voltage oscillations along the cable

Using equation S5 we can easily obtain explicit expressions for the amplitude of the voltage oscillations along the cable as a function of the cable parameters. For synchronized dendritic oscillators (i.e. $\phi = 0$) the oscillation amplitude $\bar{V}(x)$ at any point x along the cable of length l is

$$\bar{V}(x) = \bar{V}_{\text{dend}} \left| \frac{\sinh(b(L - x/\lambda)) + \sinh(b\,x/\lambda)}{\sinh(bL)} \right| \quad \text{(S15)}$$

Considering the voltage at the middle of the cable as the 'somatic' voltage $V_{\text{soma}}(t) = U(l/2,t)$, we find the somatic oscillation amplitude for synchronized oscillators:

$$\bar{V}_{\text{soma}} = \bar{V}(l/2) = \frac{\bar{V}_{\text{dend}}}{\left|\cosh(bL/2)\right|} \quad \text{(S16)}$$

Supplemental Experimental Procedures: Reduction of the full oscillator model by Fransén et al. (2004) to two variables

We describe how the full model by Fransén et al. (2004) was reduced to the two-variable model given by Equation 7 in Experimental Procedures. The gating of I_h was described by a single variable $r(V,t)$ with an activation time constant that was fitted to experimental data from Dickson et al. (2000). The I_{NaP} gating variable $n(V,t)$ was considered to be instantaneous, i.e. $n(V,t) = n_\infty(V)$, since its dynamics are much faster (~ 1 msec) than the oscillation dynamics (see also White et al., 1995; Dickson et al., 2000). Inactivation of I_{NaP} and slow activation of I_h were considered constant since their dynamics are much slower than the oscillation dynamics (Magistretti and Alonso, 1999; Dickson et al., 2000). The activation functions $n_\infty(V)$ and $r_\infty(V)$ were identical to those determined experimentally by Magistretti and Alonso (1999) and Dickson et al. (2000). Current densities were constrained by the frequency and amplitude of the resulting subthreshold oscillations recorded at the soma, which needed to be on the order of ~ 8 Hz and 1–2 mV, respectively (Alonso and Llinás, 1989; Alonso and Klink, 1993).

Supplemental Text: Stable grid fields require unrealistic membrane and input parameters to obtain sufficiently slow phase locking time constants

For stellate cells to maintain stable grid fields over many minutes – as observed in *in vivo* experiments (Hafting et al., 2005) – the phase locking time constant needs to be at least ~ 100 seconds. On the basis of our analytical results we find that this can only be accomplished by decreasing the PRC amplitude more than a factor of 1000, when considering an optimal oscillator size and including active dendritic conductances (see Figure 3 and Supplemental

Figure S3). Hence, this requires a 1000-fold increase in the total oscillation-generating current. Using the oscillator model described in Experimental Procedures as a reference, we determined the consequences of such current density increases. The model uses oscillation-generating currents based on experimental data from stellate cells. It has a similar PRC amplitude as the oscillator used in our mathematical analysis (see Supplemental Figure S2) and therefore also requires a 1000-fold increase in the current densities. The total membrane conductance of the oscillator model at the mean oscillation voltage –50 mV is ~ 0.2 mS/cm^2. Increasing the densities by a factor of 1000 gives very large dendritic conductance densities, e.g. peak g_{NaP} reaching ~ 1 nS/µm^2, similar to estimates of sodium channel densities in the axon initial segment (Kole et al., 2008). Note, however, that conductances in the axon initial segment are not persistent and so they are mostly inactivated at subthreshold potentials, while the oscillator conductances are persistently activated throughout the oscillation cycle. The increase in membrane conductance of the oscillator model to ~ 200 mS/cm^2 means that the membrane time constant decreases from 5 msec to 5 µsec. This is a 100-times faster than the (to our knowledge) fastest membrane time constants observed in neurons (0.5–1 msec in auditory brainstem neurons, see e.g. Scott et al., 2005). Furthermore, such an increase in current densities also affects the interaction between I_h and I_{NaP}, making the dynamics of the oscillator much faster and increasing its frequency substantially. Our simulations showed that a modest 3-fold increase of the oscillator current densities increased the oscillation frequency from 8 Hz (i.e. the oscillation frequency observed in stellate cells) to 18 Hz (Supplemental Figure S4D-E).

Another consequence of a very small PRC amplitude is that the synaptic input currents need to be very large to be able to affect the oscillator phase (see Supplemental Figure S1). Making a conservative estimate, the mean total synaptic conductance input at the oscillator can be at most a factor of 100 smaller than the total oscillator conductances to have an effect on the oscillator dynamics. A mean synaptic conductance density of ~ 20 mS/cm^2 requires more than 1000 excitatory synapses per 100 µm^2, assuming a mean input rate of 10 Hz, synaptic peak conductance of 300 pS, and synaptic time constant of 6 msec (Berretta and Jones, 1996). This is far beyond the observed densities of excitatory synaptic inputs in dendrites of cortical neurons, which are typically in the range of 30–50 synapses per 100 µm^2 (see, for example, Anderson et al., 1994).

Supplemental References

Anderson, J.C., Douglas, R.J., Martin, K.A., and Nelson, J.C. (1994). Map of the synapses formed with the dendrites of spiny stellate neurons of cat visual cortex. J. Comp. Neurol. *341*, 25–38.

Berretta, N., and Jones, R.S. (1996). A comparison of spontaneous EPSCs in layer II and layer IV-V neurons of the rat entorhinal cortex in vitro. J. Neurophysiol. *76*, 1089–100.

Koch, C. (1984). Cable theory in neurons with active, linearized membranes. Biol. Cyber. *50*, 15–33.

Kole, M.H., Ilschner, S.U., Kampa, B.M., Williams, S.R., Ruben, P.C., and Stuart, G.J. (2008). Action potential generation requires a high sodium channel density in the axon initial segment. Nat. Neurosci. *11*, 178–86.

Magistretti, J., and Alonso, A.A. (1999). Biophysical properties and slow voltage-dependent inactivation of a sustained sodium current in entorhinal cortex layer-II principal neurons: a whole-cell and single-channel study. J. Gen. Physiol. *114*, 491–509.

Sabah, N.H., and Leibovic, K.N. (1969). Subthreshold oscillatory responses of the Hodgkin-Huxley cable model for the squid giant axon. Biophys. J. *9*, 1206–22.

Scott, L.L., Mathews, P.J., and Golding, N.L. (2005). Posthearing developmental refinement of temporal processing in principal neurons of the medial superior olive. J. Neurosci. *25*, 7887–95.
